# Surgical unroofing of long and deep myocardial bridges in children: A 2-case report

**DOI:** 10.1097/MD.0000000000048159

**Published:** 2026-04-03

**Authors:** Fengfeng Wang, Weitao Zhang, Jintao Zhang, Xinhua Wei, Qun Li, Taibing Fan, Keming Yang

**Affiliations:** a Department of Children’s Heart Center, Fuwai Central China Cardiovascular Hospital, Zhengzhou, China; b Pediatric Cardiac Surgery Center, National Center for Cardiovascular Diseases and Fuwai Hospital, CAMS and PUMC, Beijing, China.

**Keywords:** myocardial bridge, pediatric, surgery, syncope

## Abstract

**Introduction:**

Myocardial bridge (MB) is a congenital coronary artery malformation, generally considered benign. However, we reported 2 cases of long and deep coronary MB in children and their surgical treatment.

**Patient concerns and diagnosis:**

Two children with coronary MB presented with syncope as the initial symptom. Case 1: female, 11 years old, body weight 32.5 kg. Cardiac enzymes were elevated. Electrocardiograms showed ST-T changes. Echocardiography: No abnormalities were found in cardiac function and ventricular wall motion. Coronary computed tomography angiography (CCTA) showed that the left anterior descending artery (LAD) and its branches were located deep and long within the myocardium. Coronary angiography (CAG) revealed that the LAD was nearly occluded during systole and thin during diastole, and the right coronary artery was small. Case 2: male, 12 years old, body weight 32 kg. Cardiac enzymes were elevated. Electrocardiograms showed abnormal Q waves, and the inferior and anterior ventricular walls were elevated in ST-segment. Regional wall motion abnormality and diastolic dysfunction were shown in echocardiography. CCTA showed the LAD and its branches were located deep and long within the myocardium. CAG showed that the LAD was slender in diastole and further narrowed in systole, while the right coronary artery ran short.

**Interventions:**

Both cases were given surgical MB release.

**Outcomes:**

In case 1, postoperative reexamination showed the MB was completely released, and postoperative cardiac enzymes, electrocardiograms, echocardiograms, and CCTA demonstrated the patient was gradually recovering. The 3-month follow-up found nothing abnormal detected. In case 2, partial release was performed. The LAD was located in the middle and inner part of the interventricular septum. Partial release was performed at the proximal end, while complete release was performed at the middle and distal ends. The cardiac enzymes and electrocardiograms in the 3-month postoperative follow-up examination indicated the patient recovered, while the cardiac echocardiogram revealed that there was still regional wall motion abnormality and diastolic dysfunction.

**Conclusion:**

MB in children can cause syncope and myocardial infarction. Surgical unroofing of MB is an effective clinical option. Careful preoperative examination, including CCTA and CAG, and a detailed surgical planning system are critical to the surgery.

## 1. Introduction

Myocardial bridge (MB) was first described by Reyman in 1737. It is generally regarded as a benign disease and is recommended for medical follow-up. However, some studies have shown that MB is associated with myocardial ischemia, chest pain, acute coronary syndrome, arrhythmia, and sudden cardiac death.^[1–7]^ It is recommended to classify and treat the disease based on the response to oral β-blockers and calcium channel antagonists.^[8]^ Once diagnosed, drug treatment should be attempted first.^[9]^ For patients with no obvious response to the drug treatment, surgical treatment is recommended. Although MB can occur in any epicardial artery, it is more likely to implicate the left anterior descending artery (LAD) in most cases (70%–98%).^[10]^ The surgical procedures for the MB include myocardial incision and coronary artery bypass grafting.^[11]^ Additionally, local ischemia cannot be resolved through bypass grafting surgery due to continuous compression. Currently, the relevant statistical data for symptomatic LAD surgical treatment are limited to a few studies, with a small sample size. Due to its rarity, the evidence is limited. A few studies have found that long-segment myocardial incision to relieve the MB is superior to coronary artery bypass grafting.^[12,13]^

## 2. Clinical material and method

### 2.1. The first symptoms of myocardial infarction in these 2 children were syncope following chest pain

**Figure 1.**
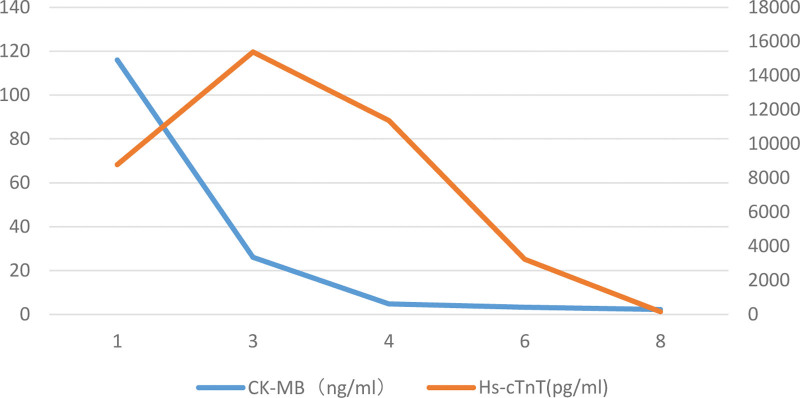
Creatine kinase isoenzyme and high-sensitivity troponin increased significantly but gradually recovered over time. CK-MB = creatine kinase-MB, Hs-cTnT = high-sensitivity cardiac troponin T.

**Figure 2.**
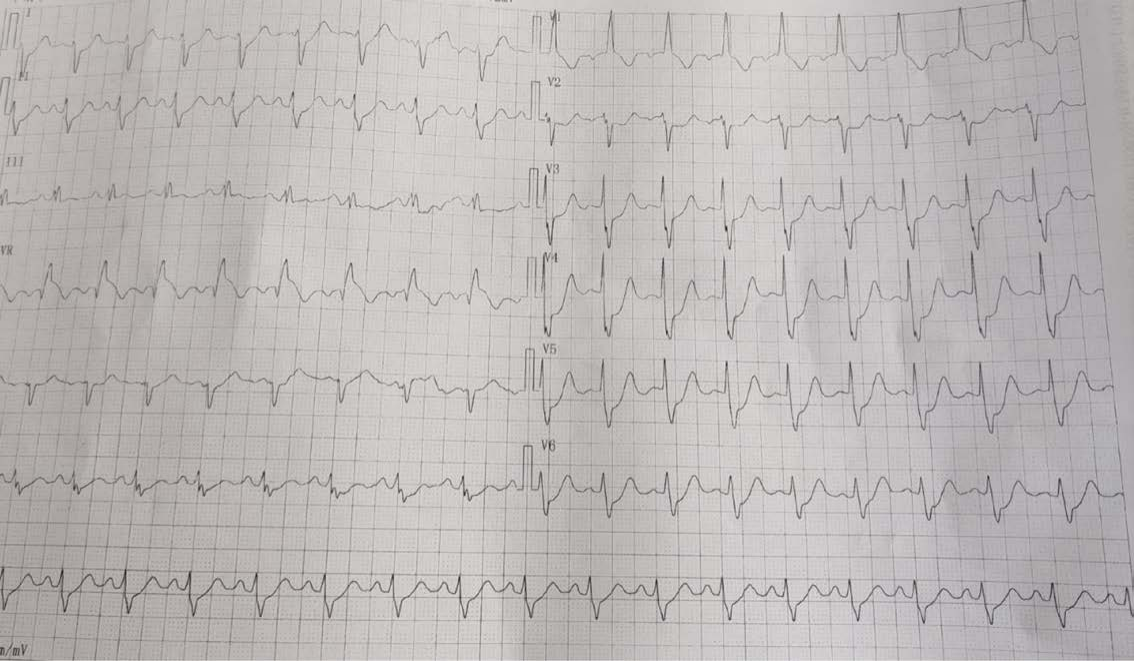
Electrocardiogram after chest pain and syncope.

**Figure 3.**
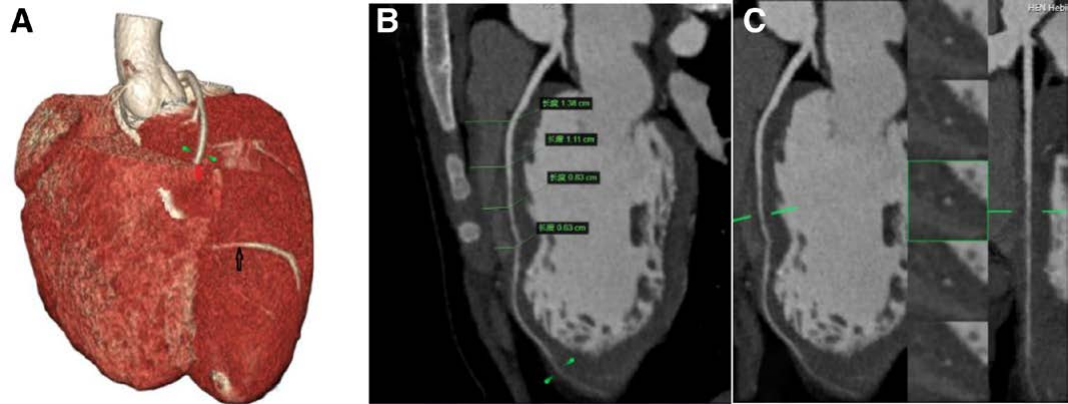
Coronary computed tomography angiography. (A) The red arrow represents the site where the myocardial bridge perforates the myocardium, and the black arrow represents the site where the myocardial bridge is close to the myocardium. (B) Represents the depth of the myocardial bridge at different locations. (C) The anterior descending coronary artery ran completely between the left and right ventricles.

**Figure 4.**
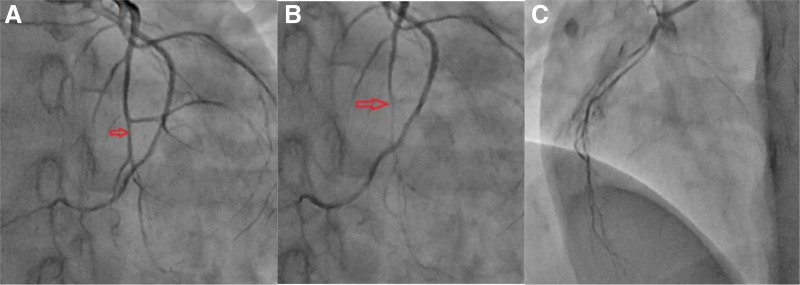
(A) Diastolic image of the heart, left anterior descending artery (the red arrow), (B) systolic image of the heart red arrow, and (C) right coronary artery.

**Figure 5.**
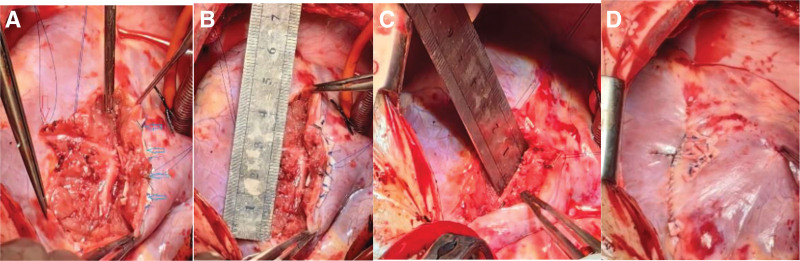
The intraoperative photo. (A) The starting point of the unroofing myocardial bridge indicated by the red arrow, and the blue arrow indicates the interrupted suture with shim after the rupture of the right ventricle. (B) Shows that the length of the myocardial bridge is 5 cm. (C) Shows that the deepest depth of the myocardial bridge is 15 mm. (D) Myocardial bridge is completely released with the epicardium sutured and compressed to stop bleeding.

Case 1 was a 11-year-old girl, weighing 32.5 kg. The initial symptom upon admission was syncope, which alleviated on its own after a few minutes. Examination revealed changes in myocardial enzymes (Fig. 1). After the onset, creatine kinase isoenzymes and high-sensitivity troponin significantly increased, and gradually recovered over time. Electrocardiogram (Fig. 2) showed ST-segment depression in the chest leads, ST-T changes in extensive leads, and complete right bundle branch block. Echocardiography demonstrated no abnormality in cardiac function and ventricular wall movement, with a coronary artery-right ventricular fistula with a shunt diameter of 1.2 mm. Coronary computed tomography angiography (CCTA) (Fig. 3) showed an MB in the LAD, characterizing the LAD and its branches running deep and long within the myocardium, and the shunt opening was superficial under the epicardium at the distal end of the second diagonal branch. Coronary angiography (CAG) (Fig. 4) suggested the LAD was thin during diastole and discontinuous during systole. Medical revascularization was difficult, and a surgical MB release surgery was performed. The intraoperative data are shown in Figure 5.

**Figure 6.**
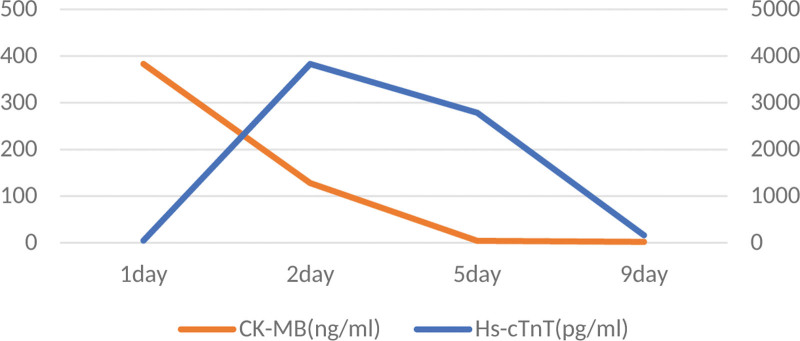
Creatine kinase isoenzyme and high-sensitivity troponin increased significantly and gradually recovered over time. CK-MB = creatine kinase-MB, Hs-cTnT = high-sensitivity cardiac troponin T.

**Figure 7.**
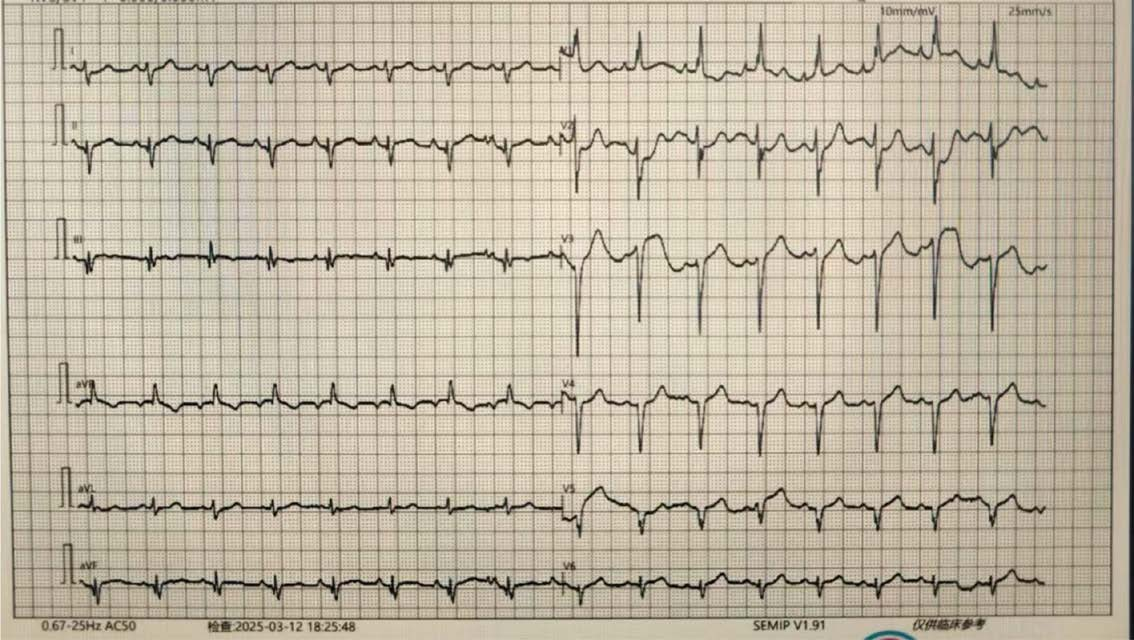
Electrocardiogram after chest pain and syncope.

**Figure 8.**
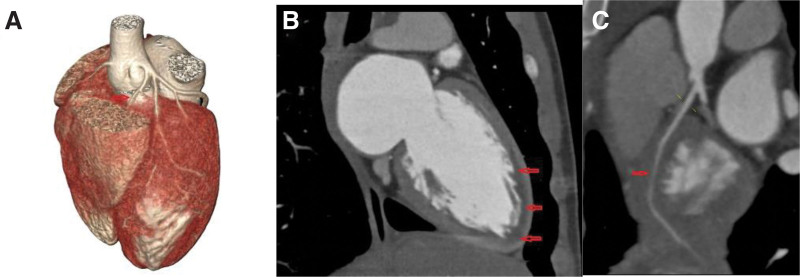
(A) Shows the myocardial bridge characterized by the left anterior descending artery and its branches all ran in the deep and long myocardium. (B) Shows diffuse low density in the left ventricle endocardium, considered as myocardial ischemia (red arrow). (C) Shows the compressed lumen of the left anterior descending artery.

**Figure 9.**
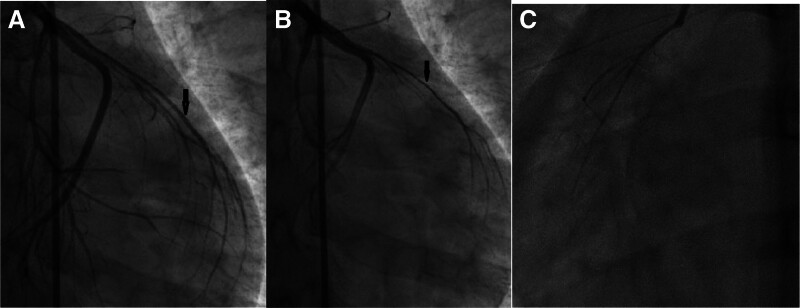
The left anterior descending artery was slender during diastole (A) and further narrower during systole (B); at the same time, the right coronary artery (C) was short and slender.

Case 2 was a 12-year-old boy, weighing 32 kg. The first symptom on admission was syncope, which relieved spontaneously after a few minutes. Myocardial enzymes (Fig. 6) were found to significantly increase after the onset of the disease, and gradually recovered over time. Electrocardiogram (Fig. 7) showed sinus rhythm and abnormal Q waves in the inferior and anterior ventricular walls along with ST-segment elevation. Echocardiography indicated regional wall motion abnormality and abnormal diastolic function. CCTA (Fig. 8A) showed the MB featuring the LAD and its branches all running in the deep and long myocardium; showed diffuse low density in the left ventricle endocardium, considered as myocardial ischemia (red arrow) (Fig. 8B), and showed the compressed lumen of the LAD (Fig. 8C). CAG results (Fig. 9A) indicated a marked slender LAD in diastole, further narrowing of LAD in systole (Fig. 9B), and the short right coronary artery (Fig. 9C).

## 3. Results

In case 1, postoperative reexamination showed the MB was completely released, and postoperative cardiac enzymes, electrocardiograms, echocardiograms, and CCTA scans demonstrated gradual recovery. Follow-up in the following 3 months showed nothing abnormal detected. In case 2, partial release was performed. The LAD was located in the middle and inner part of the interventricular septum, close to the left ventricle, with a risk of left ventricular rupture. Partial release was performed at the proximal end, and complete release was performed at the middle and distal ends. Postoperative oral medication was given. Postoperative and 3-month follow-up examination showed no abnormality in cardiac enzymes and an impressive recovery shown by electrocardiograms, while the cardiac echocardiogram revealed that there was still a regional wall motion abnormality and diastolic dysfunction of the segmental left ventricular wall (Supplemental video).

## 4. Operation

**Figure 10.**
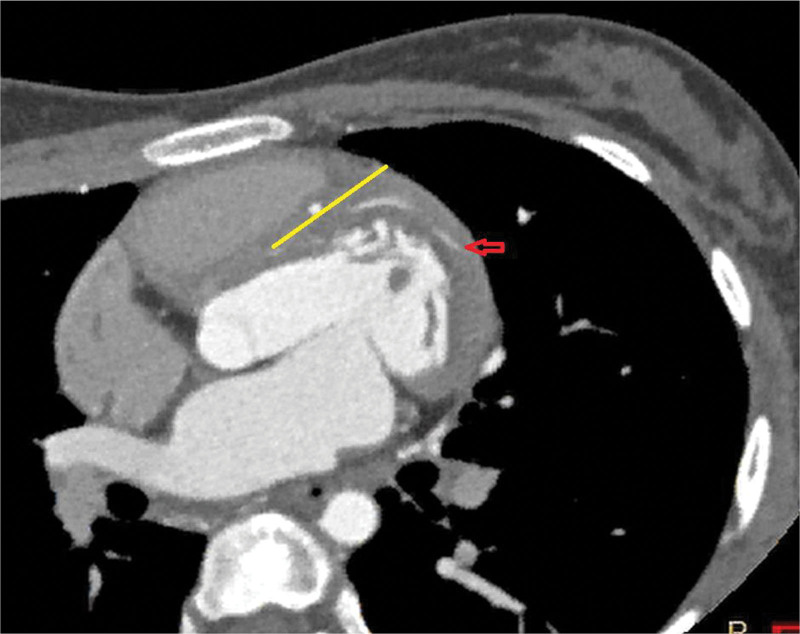
The distal end of the second diagonal branch (red arrow); the sloping ventricular septum (yellow line).

In both cases, the MB was released under general anesthesia with extracorporeal circulation. In addition, the MB is completely covered by myocardium and is thus not so easy to identify as normal coronary arteries that are exposed on the surface. As shown in Figure 3A, the middle and distal segments of the second diagonal branch were closer to the epicardium, which was more obvious in Figure 10 (red arrow); thus, this was the critical position for successful surgery.

In case 1, we chose the position in Figure 10 (red arrow) as our starting point. After blocking the myocardium, we used a surgical round blade to incise the epicardium and sharply dissect the myocardium to expose the second diagonal branch, with a depth of 2 mm. After that, we dissected along the second diagonal branch toward the proximal end, gradually and sharply cutting the myocardium layer by layer from deep to shallow until reaching the outer membrane of the coronary artery. Next, we carefully peeled it off, leaving the space between the incised myocardium more than half of the circumference of the coronary artery, thus exposing the coronary artery properly. After finding the main trunk of the LAD, we gradually and sharply dissected it toward the proximal end until reaching the left main coronary artery, where the LAD penetrates the myocardium. We used 5-0 Prolene suture with a shim for interrupted suture (Fig. 5A, blue arrow) because of the particular positions of the right ventricle and the left ventricle, and for the fear of the right ventricle rupture caused by sharp incision of the inclined interventricular septum (Fig. 10, yellow line). Then, we used the same approach to dissect along the LAD toward the distal end to completely release the MB. After the release was completed, we used 6-0 Prolene suture for continuous suture of the epicardium to compress and stop the operated tissue from bleeding.

In case 2, we applied the method of choosing 2 positions as the starting points of the surgery to release the MB. At first, we chose to release the bridge from distal to proximal. The fact that the proximal end was too deep and close to the left ventricle and excessive incision of the ventricular septum may damage the left ventricular and even risk ventricular rupture made us start the release from the proximal end toward the distal end, and then we cut the pulmonary artery transversely. Even if both ends were started simultaneously, it was not possible to completely release it. At the middle and proximal segments of the LAD, about 1.5 cm of the myocardium was not released completely. We used the same method to suture the epicardium.

## 5. Discussion

There was no guideline for the diagnosis and treatment of MB, especially for children. Medical therapy is needed, and β-blockers are important in the first-line treatment for such patients.^[14]^ The drug favorably inhibits sympathetic excitability, thereby reducing heart rate, increasing relaxation time, and allowing muscle bridge decompression. Ivabradine is more effective when combined with calcium channel blockers.^[9]^ Daba et al^[2]^ indicate that MB is 1 of the causes of myocardial infarction (MI). According to the retrospective literature reviews, MB can lead to serious complications after MI, including arrhythmia, acute coronary syndrome, and sudden death Similarly, Maeda et al^[15]^ reported the cases where 14 children with MB were treated with incision and release at Stanford University Medical Center, and the postoperative results were satisfactory. Boyd et al^[12]^ also had the same opinion and recommended active surgical unroofing of hemodynamically significant MB in the pediatric population. Hostiuc et al^[16]^ made a detailed analysis of 3 large database sets, concluding that MB was associated with major cardiovascular events. In light of the actual situation where the patients in the 2 cases suffered syncope as the first symptom, whose MB ran deep and long, and accordingly, the computed tomography and digital subtraction angiography showed obvious compression of the LAD or even devascularization, we chose to perform the MB release on them under general anesthesia with extracorporeal circulation. Sakamoto et al^[17]^ reported that preoperative and postoperative evaluation of CCTA was very crucial for surgical decision-making. The selection of preoperative surgical methods and the evaluation of postoperative surgical effects were critical for the author to find the starting point of coronary artery incision. In the 2 cases, the biggest challenge of the operation was not the release technique of MB, but the selection of the starting point of MB release, trying not to cut into the ventricle, especially the left ventricle, which would cause postoperative cardiac rupture. In case 1, we evaluated the location of the starting point of the surgical incision on CTA. In case 2, we performed a proximal transection of the pulmonary artery and positioned the left main coronary artery and LAD with absolute accuracy. During the dissection, it was found that the distance from the LAD to the left ventricle was about 5 mm, running deep and very close. Postoperative echocardiography, myocardial enzymes, and electrocardiogram showed significant improvement. By contrast, the medical follow-ups in the following 4 years of adults with MB who did not undergo surgeries reported no serious heart problems in the study by Cicek et al,^[10]^, with only 30% to 50% stenosis of the coronary artery during myocardial contraction, and no major adverse cardiac events occurring with oral drug treatment alone. Long-term follow-up of MB is recommended in both adults and children.^[18]^ Agrawal et al^[19]^ suggested preoperative use of coronary intravascular ultrasound to diagnose the condition of stratification of coronary MB in children. When the intravascular ultrasound showed that the lumen diameter of the inner segment of the heart was reduced by ≥70% in systole and continued to decrease by ≥35% in mid-late diastole, it is time for active intervention. In this study, the preoperative CAG of 2 children showed that the systolic significant stenosis was ≥70% and needed active treatment. To effectively diagnose and treat MB in children, Erol^[20]^ suggested that it was necessary to conduct multicenter, prospective design and long-term follow-up studies on children with MB in centers with a large number of cases, and formulate the criteria for the diagnosis and treatment of children with MB based on such studies to make the diagnosis and treatment of such cases more accurate and efficient.^[21]^ Evbayekha et al^[22]^ mentioned in their review that, in view of the particularity of MB anatomy, some MB that could not be completely released needed long-term follow-up and standardized drug treatment; otherwise, the long-term effect may not be ideal. It is suggested that appropriate diagnostic mode, effective treatment, and long-term follow-up are vital to reduce MB-related morbidity and mortality. MB combined with coronary atherosclerosis will significantly affect the long-term results,^[23,24]^ so it is recommended that patients with MB should be actively intervened in advance to prevent coronary atherosclerotic heart disease. In 2024, the European Society of Cardiology^[25]^ recommended patient-centered, drug therapy–based, comprehensive treatment for nonobstructive coronary artery disease once the diagnosis of invasive coronary function monitoring was confirmed. Tarantini et al^[11]^ proposed that the surgical standard of MB in adults should be longer than 25 mm and deeper than 5 mm.

## 6. Conclusion

MB in children can cause syncope and MI. Surgical unroofing of the MB is an effective surgical option. Careful preoperative examination of CCTA and CAG, and a detailed surgical planning system are key to the surgery.

## 7. Limitations

This study is limited due to a small sample size, short follow-up time, and selection bias. A longer follow-up is needed to determine proper treatment, particularly in patients who initially present with cardiogenic syncope. A larger sample size and longer follow-up time will further promote our comprehensive understanding of the natural and postoperative course of MB patients.

## Author contributions

**Investigation:** Weitao Zhang.

**Formal analysis:** Jintao Zhang.

**Supervision:** Xinhua Wei, Qun Li.

**Visualization:** Taibing Fan.

**Writing – original draft:** Fengfeng Wang.

**Writing – review & editing:** Keming Yang.
